# Comprehensive analysis of T cell leukemia signals reveals heterogeneity in the PI3 kinase-Akt pathway and limitations of PI3 kinase inhibitors as monotherapy

**DOI:** 10.1371/journal.pone.0193849

**Published:** 2018-05-25

**Authors:** Olga Ksionda, Marsilius Mues, Anica M. Wandler, Lisa Donker, Milou Tenhagen, Jesse Jun, Gregory S. Ducker, Ksenia Matlawska-Wasowska, Kevin Shannon, Kevan M. Shokat, Jeroen P. Roose

**Affiliations:** 1 Department of Anatomy, University of California San Francisco, San Francisco, California, United States of America; 2 Department of Pediatrics, University of California San Francisco, San Francisco, California, United States of America; 3 Department of Molecular Pharmacology, Howard Hughes Medical Institute, University of California San Francisco, San Francisco, California, United States of America; 4 Department of Pediatrics, Division of Hematology-Oncology, University of New Mexico, Health Sciences Center, Albuquerque, New Mexico, United States of America; University of Navarra, SPAIN

## Abstract

T cell acute lymphoblastic leukemia (T-ALL) is an aggressive hematologic cancer. Poly-chemotherapy with cytotoxic and genotoxic drugs causes substantial toxicity and more specific therapies targeting the underlying molecular lesions are highly desired. Perturbed Ras signaling is prevalent in T-ALL and occurs via oncogenic *RAS* mutations or through overexpression of the Ras activator RasGRP1 in ~65% of T-ALL patients. Effective small molecule inhibitors for either target do not currently exist. Genetic and biochemical evidence link phosphoinositide 3-kinase (PI3K) signals to T-ALL, PI3Ks are activated by Ras-dependent and Ras-independent mechanisms, and potent PI3K inhibitors exist. Here we performed comprehensive analyses of PI3K-Akt signaling in T-ALL with a focus on class I PI3K. We developed a multiplex, multiparameter flow cytometry platform with pan- and isoform-specific PI3K inhibitors. We find that pan-PI3K and PI3K γ-specific inhibitors effectively block basal and cytokine-induced PI3K-Akt signals. Despite such inhibition, GDC0941 (pan-PI3K) or AS-605240 (PI3Kγ-specific) as single agents did not efficiently induce death in T-ALL cell lines. Combination of GDC0941 with AS-605240, maximally targeting all p110 isoforms, exhibited potent synergistic activity for clonal T-ALL lines *in vitro*, which motivated us to perform preclinical trials in mice. In contrast to clonal T-ALL lines, we used a T-ALL cancer model that recapitulates the multi-step pathogenesis and inter- and intra-tumoral genetic heterogeneity, a hallmark of advanced human cancers. We found that the combination of GDC0941 with AS-605240 fails in such trials. Our results reveal that PI3K inhibitors are a promising avenue for molecular therapy in T-ALL, but predict the requirement for methods that can resolve biochemical signals in heterogeneous cell populations so that combination therapy can be designed in a rational manner.

## Introduction

T cell acute lymphoblastic leukemia (T-ALL) is an aggressive hematologic cancer characterized by abnormal growth and proliferation of early T cell progenitors. T-ALL affects children and adults, and despite substantial progress with modern chemotherapy, the prognosis remains poor for patients who relapse. Currently, the 5-year overall survival rate is approximately 80% for children with T-ALL and 45% for adults [1]. Modern treatment regimens consist of poly-chemotherapy with cytotoxic and genotoxic drugs that both cause substantial acute toxicity and are associated with a variety of late adverse health effects [2]. More specific and less toxic therapies are therefore desired to improve disease outcomes and quality of life both during and after treatment. One attractive strategy for achieving this goal is to target aberrant signal transduction pathways that drive the growth and survival of leukemic blasts.

The Notch pathway is an attractive candidate for targeted inhibition, since more than half of all T-ALLs harbor mutations in this pathway [3]. However, the dose levels of a “first generation” Notch inhibitor was limited by gastrointestinal toxicity and this agent had limited clinical efficacy [4]. Hyperactive Ras signaling is another attractive candidate target in T-ALL. Oncogenic Ras signals are observed in ~65% of T-ALL patients [5, 6], either via an oncogenic *RAS* mutation, inactivation of the *NF1* tumor suppressor, or overexpression of the activator Ras guanine nucleotide releasing protein 1 (RasGRP1) [7, 8]. As effective small molecule inhibitors of either Ras or RasGRP1 do not currently exist, we focused on class I phosphoinositide 3-kinases (PI3Ks), which are activated by Ras-dependent and Ras-independent mechanisms [9–15]. When in its active, GTP-bound conformation, Ras binds to and activates PI3K at the plasma membrane, and PI3K’s role in oncogenic cell growth and protein translation makes it an attractive candidate for targeted anti-cancer therapy [16]. PI3Ks are divided into four classes based on their structures and substrate specificities. Members of class I, the focus here, are heterodimeric proteins consisting of regulatory and catalytic subunits. Upon activation, class I PI3Ks produce PI3P, PI(3,4)P_2_ and PI(3,4,5)P_3_ (Phosphatidylinositol (3,4,5)-trisphosphate). PI(3,4,5)P_3_ serves as a plasma membrane anchor for cytoplasmic proteins such as PDK1 and Akt that signals to promote cell cycle progression and growth. Inhibitors that interfere with the PI3K pathway are currently being evaluated in many clinical trials (Clinicaltrials.gov).

Almost 50% of pediatric T-ALL patients harbor mutations in genes encoding protein components of the PI3K-Akt pathway including *PIK3CA*, *AKT*, and *PTEN*, a major negative regulator of the pathway [17, 18]. While T-ALL cells frequently activate the Ras-PI3K pathway, there is marked heterogeneity across different leukemias and this question has not been analyzed systematically [6, 17, 19–21]. *In vitro* data from human T-ALL cell lines suggest that inhibition of PI3K may have anti-leukemic effects [21–24]. However, there are only limited *in vivo* data [21, 25] and results from studies of isoform-specific PI3K inhibitors have been conflicting [24, 26]. To better assess PI3K inhibitors as a potential therapeutic strategy for T-ALL, we performed comprehensive analyses of PI3K-Akt signaling in T-ALL cells using multiplex, multiparameter flow cytometry with pan- and isoform-specific PI3K inhibitors, tested the effect of candidate compounds to induce cell death and inhibit proliferation, and performed preclinical mouse trials with pan- and γ-isoform-specific PI3K inhibitors. Our results demonstrate that combination therapy with PI3K inhibitors is a promising avenue for future molecular therapy but also warn that thorough studies with high-resolution methods are required to fully resolve complex biochemical signals in heterogeneous cell populations of T-ALL.

## Results

### T-ALL cells with high PI3K-Akt signals

**Fig 1:**
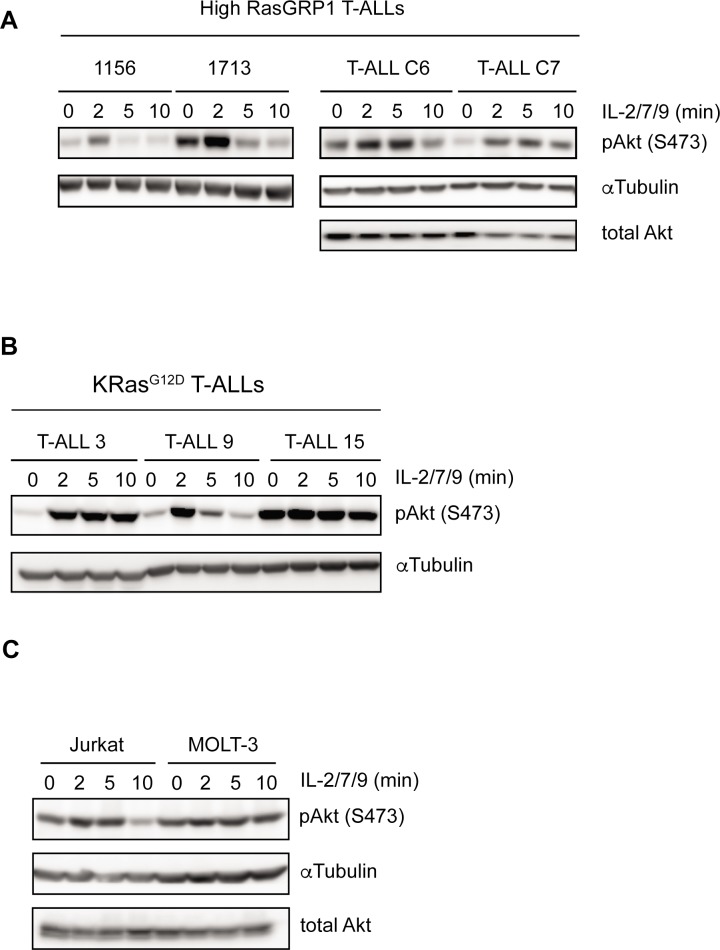
PI3K-Akt signals in T cell leukemia are affected by doxorubicin treatment. **A-C.** Western blot analysis of basal and cytokine-induced p-Akt (S473) in mouse (A-B) and human T-ALL cell lines (C). 1156, 1713, T-ALL C6 and C7 are mouse T-ALL cell lines with leukemia virus insertions in *RasGRP1* causing RasGRP1 overexpression (A). T-ALL 3, T-ALL 9 and T-ALL 15 are mouse T-ALL cell lines which harbor a KRas^G12D^ mutation. Representative blots of three or more (A & B) and two (C) independent experiments. Blotting for total Akt or αTubulin serves to demonstrate equal loading.

T-ALL cells characterized by either endogenous oncogenic KRas^G12D^ expression or by over-expression of the Ras activator RasGRP1 generate two distinct oncogenic Ras signals [6]. We selected murine T-ALL cell lines characterized by these distinct types of Ras signaling for Western blot analysis with an antibody that detects phosphorylation on serine 473 (S473) of Akt as a proxy for PI3K pathway activation at baseline. We also treated cells with a cocktail of cytokines important for T-ALL proliferation *in vivo* (reviewed in (7)) to investigate the magnitutie of cytokine-induced PI3K-Akt signaling. We observed widespread phosphorylation of Akt (pAkt) in T-ALL cells with either RasGRP1 overexpression or an oncogenic *Kras* mutation and in human Jurkat and MOLT-3 T-ALL cell lines, yet with heterogeneous intensity and different patterns of activation either at baseline and/or following cytokine stimulation (**Fig 1A**–**1C**). These results expand on previous findings in human T-ALL cell lines [6, 17, 19–21] and further motivated us to perform a comprehensive analysis of PI3K-Akt signaling in T-ALL.

### A high throughput PI3K-Akt phospho-flow platform and PI3K isoforms in T-ALL patients

**Fig 2:**
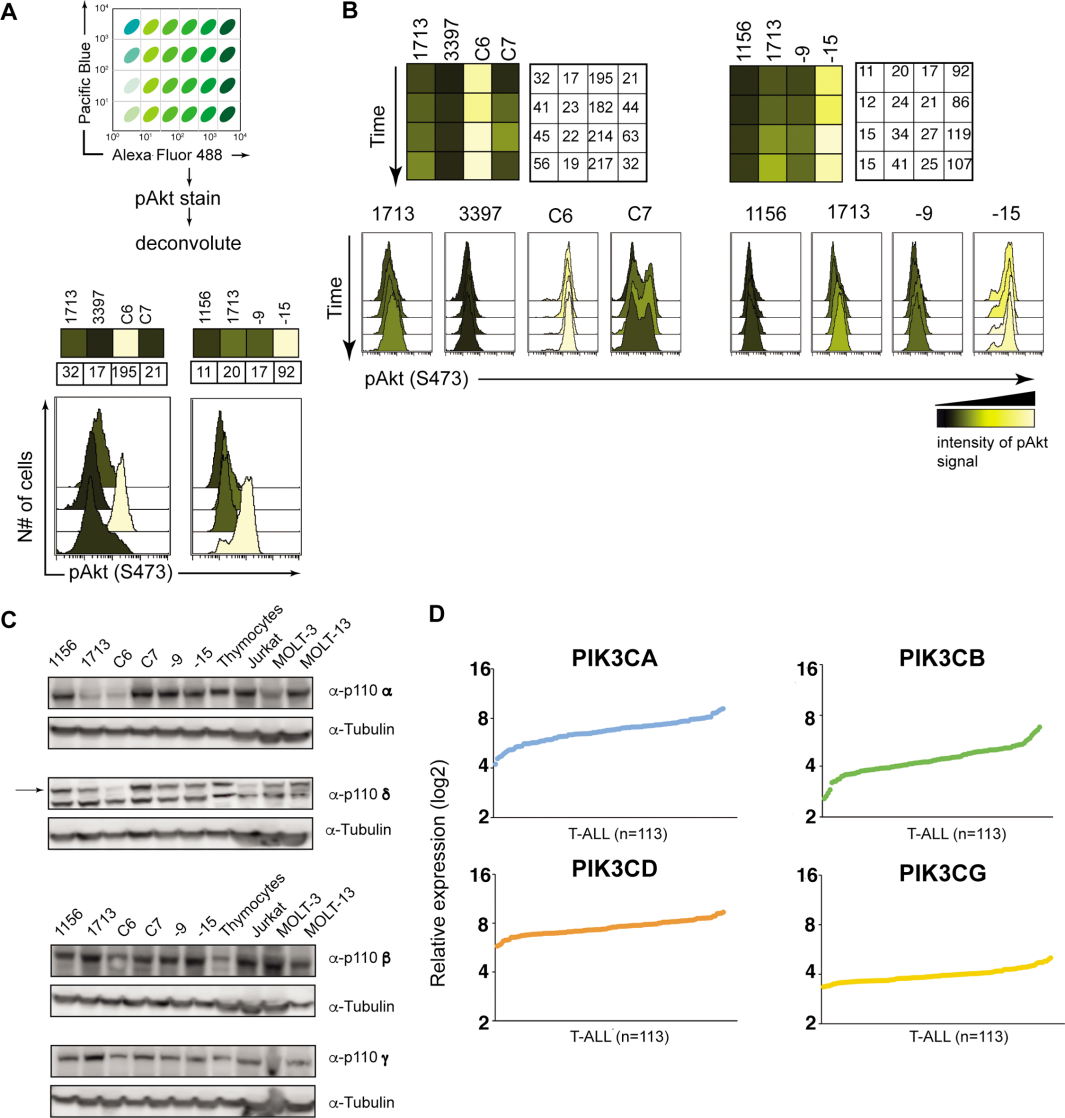
PI3K-Akt signals are heterogeneous in T-ALL cell lines. **A.** Scheme showing fluorescent cell barcoding of 24 samples and pAkt staining. Heatmaps and histograms showing basal levels of phospho-Akt (S473) in a panel of murine T-ALL cell lines overexpressing RasGRP1 (T-ALLs 1156, 3397, C6, C7) or harboring a Kras^G12D^ mutation (T-ALL 9 and 15). Numbers indicate geometric mean for phospho Akt signal. **B.** Heatmaps and histograms showing phospho Akt levels (S473) in T-ALL cell lines after stimulation with IL-2/7/9 cytokines. Each row corresponds to a 0, 2, 5 and 10 minute time point. Numbers indicate geometric mean for phospho Akt signal. Representative flow assays from at least three experiments. **C.** Western blot analysis of p110 catalytic subunit expression in murine (1156 through T-ALL 9) and human (Jurkat, MOLT-3 and MOLT-13) T-ALL cell lines. Murine thymocytes (from C57BL/6 mouse) served as a positive control (n = 2). **D.** Standard Affymetrix analysis was used to generate and normalize signal intensities of mRNA expression for four p110 catalytic subunits in samples from 113 pediatric T-ALL patients treated on COG studies 9404 and AALL0434. Values were RMA-normalized.

To comprehensively examine PI3K-Akt signaling in T-ALL, we developed a high throughput platform with fluorescent cell barcoding (FCB) coupled to phospho-flow. FCB with different concentrations of Pacific Blue and Alexa Fluor 488 creates a 6x4 matrix that allows us to stain, acquire, and analyze 24 samples in one tube followed by deconvolution (**Fig 2A**). Intracellular staining of baseline pAkt (S473) levels by phospho-flow in unstimulated T-ALL cells followed by Cytobank software analysis generated heat maps, which revealed the same heterogeneity in baseline pAkt levels as seen in Figs 1 and (**2A**). Using this platform, we also confirmed heterogeneous changes in pAkt levels in T-ALL cell lines after cytokine stimulation (**Fig 2B**).

The four catalytic subunits of PI3K differ in their tissue distribution: PI3Kα and β are expressed ubiquitously whereas expression of the δ and γ isoforms is restricted to immune cells [27]. According to the Immgen database, T cells and their precursors express all four isoforms; p110α, β, δ, and γ. Western blot analysis confirmed that all murine and human T-ALL cell lines tested expressed these four isoforms at comparable levels. Levels were also roughly similar to those observed in normal murine thymocytes (**Fig 2C**). An analysis of 113 pediatric T-ALL patient samples confirmed similar expression levels of all four isoforms with little variation between patients (**Fig 2D**). Interestingly, we observed a modest inverse correlation between RasGRP1 expression and the expression of p110α, β, and δ, but not γ (**S1A Fig**). We previously reported that T-ALL patients with high RasGRP1 expression responded relatively well to treatment (**S1B Fig** and [6]) and also find that the lymphoblasts of patients in this dataset who failed induction therapy express higher expression of p110 β or δ isoforms (**S1C Fig)**. The universal expression of p110α, β, δ, and γ in T-ALL highlights the need to understand the role of all isoform-specific signaling events in order to design effective therapeutic interventions.

### Pan-PI3K inhibitors but also PI3Kγ-specific compounds extinguish all Akt signals

**Fig 3:**
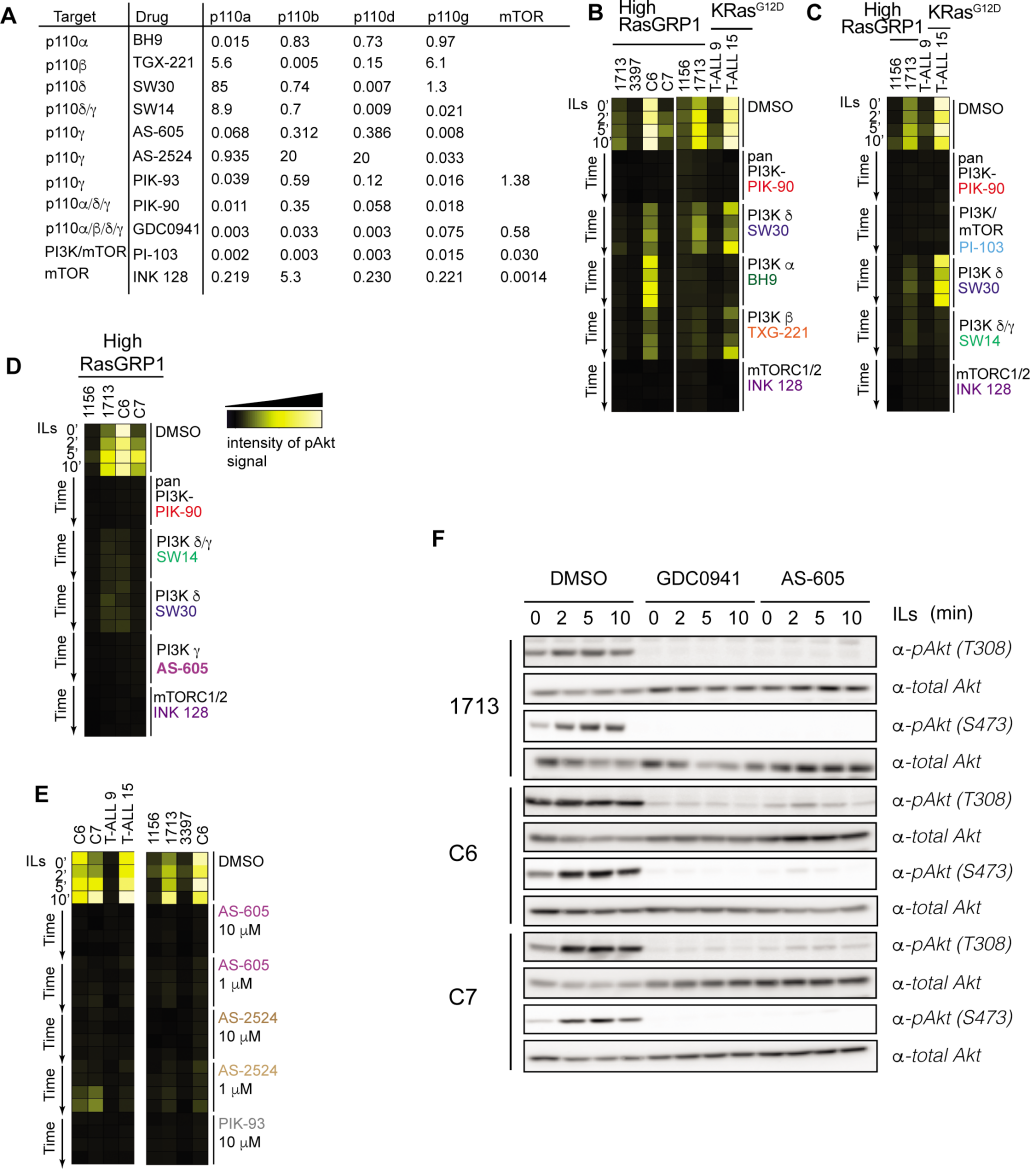
AS-6052425, a PI3Kγ inhibitor, effectively blocks phospho-Akt expression in T-ALL cell lines. **A.** IC50 values (μM) of compounds used in this study for major targets. Source: Selleckchem.com **B-E**. Heatmaps of phosho-Akt (S473) signals in T-ALL cell lines (columns) stimulated with cytokines for 0, 2, 5 or 10 minutes (rows). Cells were treated with indicated drugs (at 10 μM unless otherwise indicated) or vehicle for 30 minutes prior to stimulation. Representative heatmaps from at least three experiments. **F**. Western blot analysis of phospho-Akt (T308 and S473) signals in 1713, C6 and C7 T-ALL cell lines treated with 10 μM GDC0941 (pan-PI3K inhibitor), 10 μM AS-605 (PI3K γ specific inhibitor) or vehicle (DMSO) and stimulated with cytokines IL-2/7/9. Representative blot from at least two experiments. Blotting for total Akt serves to demonstrate equal loading.

We next assessed the effects of using available small molecule compounds to inhibit PI3K-Akt signaling in T-ALL cell lines. The IC50 values of each PI3K and mTOR inhibitor used in this study are shown in **Fig 3A**. The mTOR inhibitor serves as a positive control here since mTORC2 feeds directly into phosphorylation of Akt at S473 [28]. We observed uniform inhibition of pAkt across all T-ALL cell lines using the pan-PI3K inhibitor PIK-90, the dual PI3K/mTOR inhibitor PI-103, and the mTOR inhibitor INK 128 (**Fig 3B**–**3D**). The degree of pathway inhibition in T-ALL cells exposed to more selective PI3K p110α, β, and δ inhibitors was more heterogenenous (**Fig 3B**–**3D**) and supports the idea that different leukemias show variable dependence on each PI3K isoform to activate Akt. Surprisingly, the PI3K p110γ inhibitor AS-605 (AS-605240), blocked all Akt phosphorylation in 1156, 1713, C6, and C7 cell lines with high RasGRP1 expression (**Fig 3D**). PI3Kγ can bind to activated Ras but is also recruited to the membrane via adapters that couple to receptor tyrosine kinases or G protein-coupled receptors [14, 15]. To confirm these findings and rule out possible off-target effects of AS-605, we also tested structurally distinct PI3K γ-specific inhibitors AS-2524 (AS-252424) and PIK-93. All three compounds achieved a similar, durable biochemical inhibition of the PI3K-Akt pathway in multiple cell lines including KRas^G12D^–harboring T-ALL9 and T-ALL15 (**Fig 3E**). Lastly, we confirmed our phospho-flow data with traditional Western blot analysis. This method allowed us to assess phosphorylation of Akt on threonine 308, another activating phosphorylation event that cannot be detected by flow cytometry with the available pAkt (T308) antibodies. Treatment with AS-605 effectively suppressed phosphorylation of Akt at both sites, T308 and S473. In addition, the degree of inhibition was comparable to that observed in response to treatment with the pan-PI3K inhibitor GDC0941 (Pictilisib), a compound in advanced clinical development (**Fig 3F**). In summary, only pan-PI3K inhibitors like PIK-90 and GDC0941 and selective PI3Kγ inhibitors like AS-605 and AS-2524 effectively reduce baseline and cytokine-induced PI3K-Akt signals in T-ALL cell lines.

### AS-605 and GDC0941 as single agents cause only limited cytotoxicity

**Fig 4:**
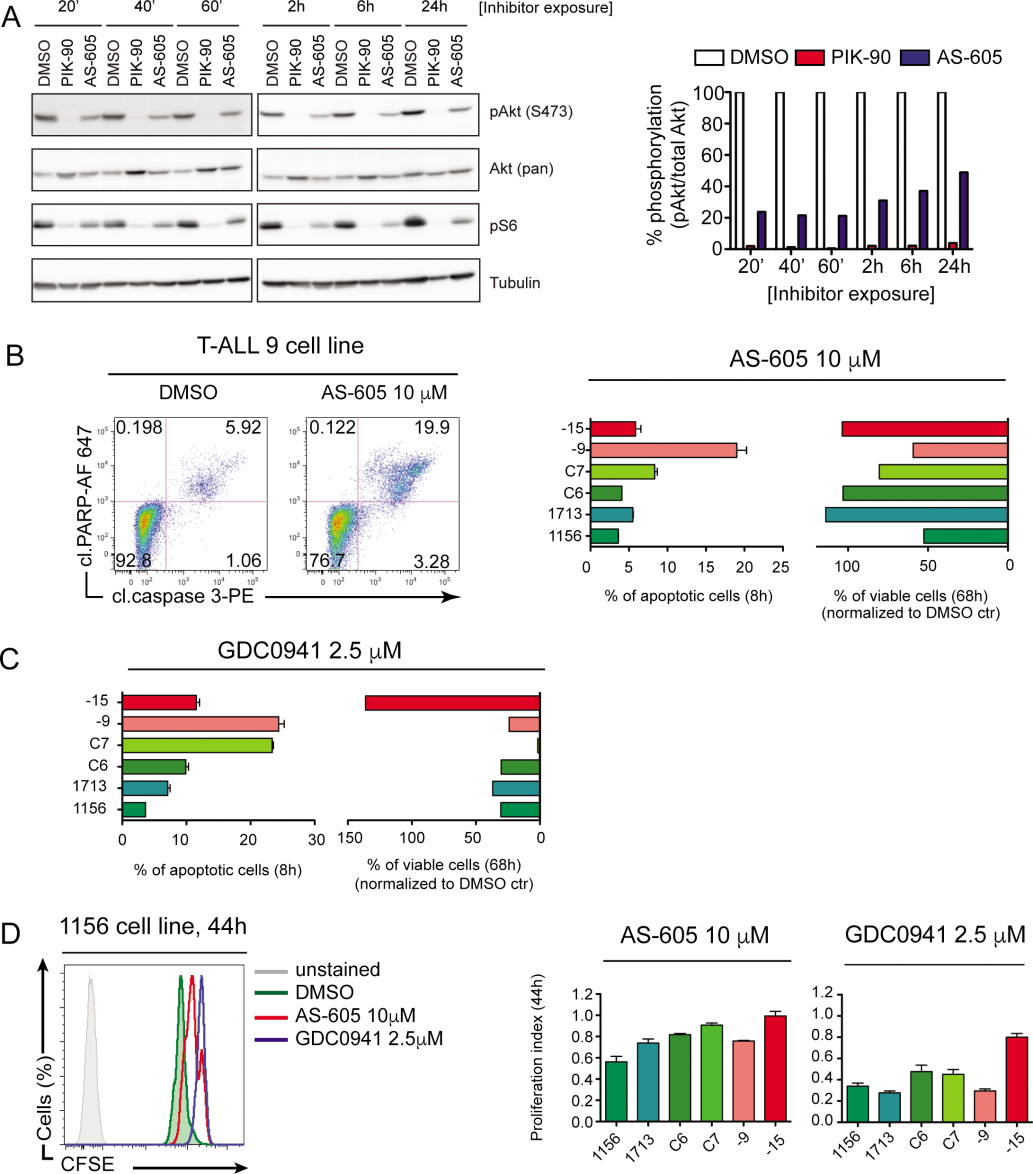
Pan- and γ-specific PI3K inhibitors have limited cytostatic effects on T-ALL cells. **A.** (Left) Western blot analysis of phospho Akt (S473) and phospho pS6 signals in C6 T-ALL cell line treated with 10 μM PIK90 (pan-PI3K inhibitor), 10 μM AS-605 (PI3K γ-specific inhibitor) or vehicle control (DMSO) for indicated times. (Right) Quantification of the Western blot shown on the left. The intensity of pAkt signal was normalized against total Akt and DMSO control was set to 100% for each time point. **B-C** Representative FACS dot plots showing gating of apoptotic cells that are double positive for cleaved caspase-3 and cleaved PARP. Left bar graph shows induction of apoptosis by AS-605 (B) or GDC0941 (C) after 8 hours of treatment. Graph on the right shows the percentage of viable cells after 68 hours of inhibitor treatment. Data were normalized to DMSO control, which was set as 100%. One representative example out of three independent experiments is shown. Error bars are SD of duplicates. **D.** Histogram showing CFSE dilution of 1156 T-ALL cell line treated with AS-605, GDC0941 or vehicle control 44 hours after drugs were added. The effect of drugs on proliferation was measured by calculating proliferation index (ratio of geometric mean of DMSO over drug treated samples). Shown is mean of two or three experiments ±SD.

We assessed the longer-term effects of PIK-90 and AS-605 treatment on Akt phosphorylation (S473) as a proxy for PI3K pathway activation, and phosphorylation of the ribosomal protein S6 (pS6) as a proxy for the mTORC1-S6K-S6 pathway. Both compounds effectively reduced pAkt and pS6 levels, but PIK-90 lead to more profound biochemical inhibition (**Fig 4A**). We next sought to determine whether achieving prolonged biochemical inhibition of these pathways would result in apoptosis or block proliferation since the PI3K/Akt pathway is known to promote both cell survival and growth [29]. To address this question, we measured apoptosis by staining for cleaved caspase-3 and cleaved PARP (hallmarks of early apoptosis) after eight hours, and cell viability by DAPI dye-exclusion at 68 hours. Despite inhibiting Akt phosphorylation, AS-605 induced only modest levels of apoptosis after eight hours of treatment. Moreover, AS-605 also had limited efficacy at a later time point (68-hours) (**Fig 4B**). GDC-0941, a pan-PI3K inhibitor like PIK-90, induced similarly modest levels of apoptosis but a more pronounced decrease in viability after 4 days in culture, albeit to varying degrees in different T-ALL cell lines (**Fig 4C and 4D**). Thus, effective biochemical inhibition of PI3K-Akt signals fails to efficiently induce death in cultured T-ALL cell lines.

### Targeting PI3K *p110 α β*, *δ and γ* isoforms with GDC0941/AS-605 combination treatment

**Fig 5:**
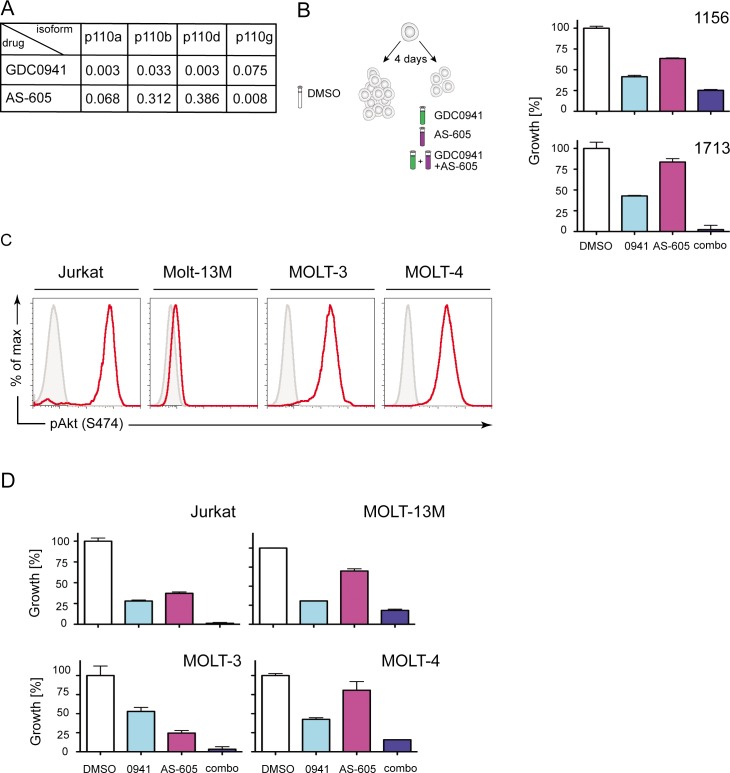
Combination therapy with GDC0941 and AS-605 demonstrates *in vitro* efficacy. **A.** Table of IC50 values for GDC-0941 and AS-605 showing effect on different PI3K isoforms. Source: Selleckchem.com **B.** Cells were seeded in the presence of each inhibitor or the combination. Bar graphs show the effect of combining GDC0941 and AS-605 inhibitors on growth of 1156 and 1713 T-ALL cell lines. Cell counts of the DMSO-treated control were set at 100% and the effect of each of treatment was calculated as a proportion of that. **C.** Histograms showing baseline phospho-Akt (S473) levels in human T-ALL cell lines (red line). Grey-filled histograms depict unstained controls. **D.** Bar graphs showing the effect of combining GDC0941 and AS-605 inhibitors on growth of human T-ALL cell lines. As in (B) cell counts of the DMSO treated control were set at 100% and the effect of each of treatment was calculated as a proportion of that.

Because GDC0941 is a relatively poor PI3K γ inhibitor (**Fig 5A**), we sought to efficiently target all four p110 isoforms by combining GDC0941 and AS-605 (**Fig 5B**). Indeed, exposing T-ALL cell lines 1156 and 1753 to both drugs profoundly inhibited their growth (**Fig 5B**). Despite substantial heterogeneity in pAkt levels (**Fig 5C),** the growth of human T-ALL cell lines was also markedly reduced in response to the GDC0941/AS-605 combination (**Fig 5D**). This encouraging result prompted us to conduct a preclinical trial in an established mouse model of T-ALL to test this treatment combination.

### Preclinical testing of GDC0941/AS-605 combination treatment in T-ALL

Mouse cancer models generated using insertional mutagenesis (IM) recapitulate the multi-step pathogenesis and inter- and intra-tumoral genetic heterogeneity that is a hallmark of advanced human cancers. We previously performed retroviral insertional mutagenesis in mice to generate primary T-ALLs that we subsequently transplanted into sub-lethally irradiated recipient mice to conduct preclinical trials of targeted anti-cancer drugs, including GDC0941 [25, 30]. In this system, the maximally tolerated dose (MTD) of GDC0941 is 125 mg/kg/day as a single agent and 100 mg/kg/day when given together with the MEK inhibitor PD0325901 [25]. We administered AS-605 at a maximum dose of 50 mg/kg/day based on published data [31]. Sub-lethally irradiated mice treated with this dose of AS-605 as a single agent, with GDC0941 (100 mg/kg/day) alone, or with both drugs for four weeks exhibited no obvious toxicity as determined by examination of organs and complete blood cell counts (CBCs) (**S2 Fig**).

**Fig 6:**
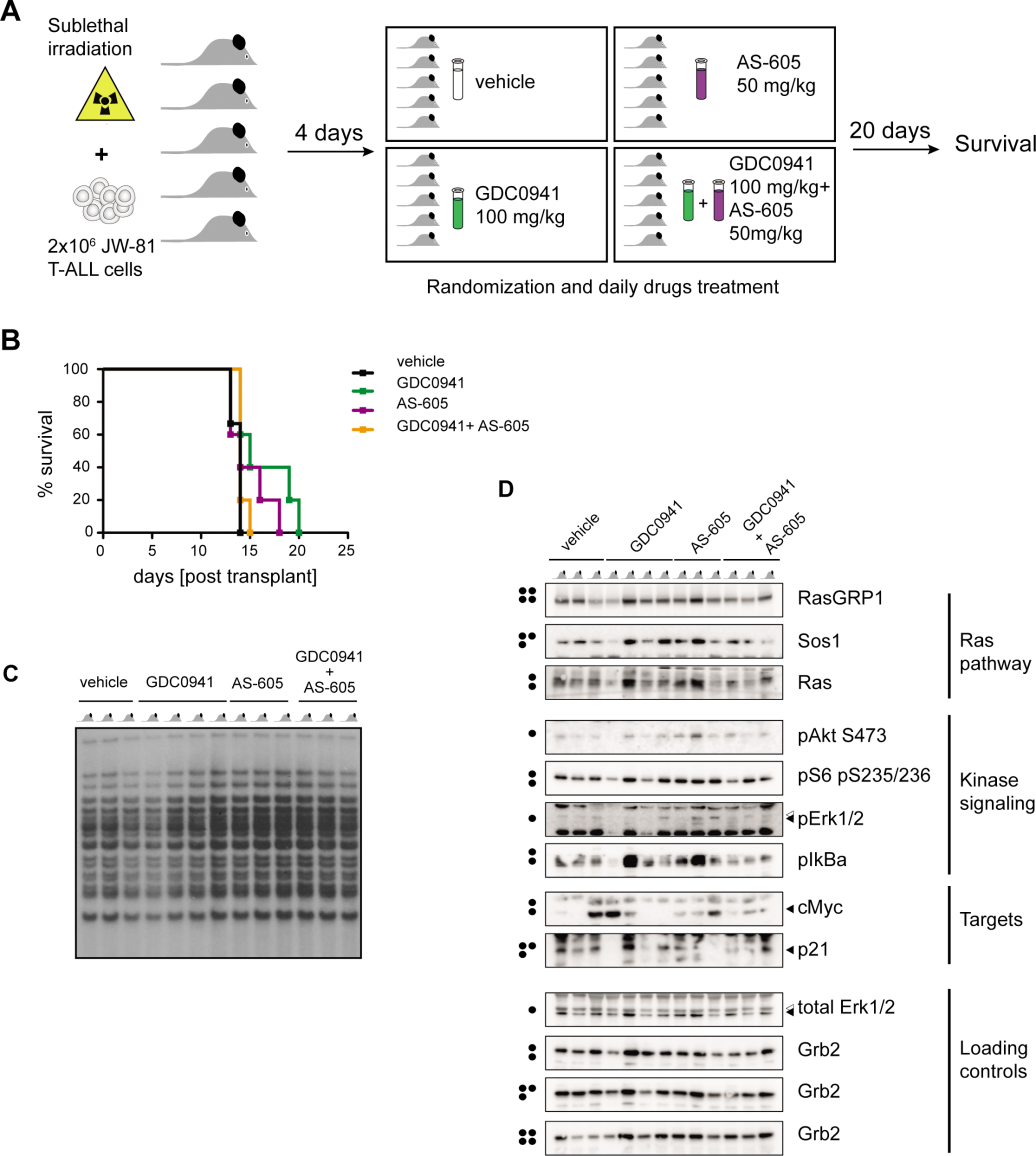
Combining GDC0941 and AS-605 inhibitors does not show *in vivo* efficacy. **A.** Diagram showing preclinical trial set-up. Four days after transplant of primary JW-81 T-ALL cells, mice were randomized into four treatment arms: vehicle control, GDC0941, AS-605, or GDC0941 + AS-605. Three mice were enrolled on the vehicle control arm and five mice were enrolled on each treatment arm. **B.** Kaplan-Meier graph showing percentage of mice surviving treatment with PI3K inhibitors as a function of time. Curves are not significantly different (Mantel-Cox test). Median survival was 14 days post-transplant for vehicle control, AS-605 and GDC0941+AS-605 and 15 days for GDC0941-treated mice. **C.** Southern blot of analysis of MOL4070 integration sites in bone marrow from 13 T-ALL samples from the four preclinical trial arms. Note that no new bands are present in samples harvested from treated mice. Each lane represents bone marrow from one animal. **D.** Western blot analysis of bone marrow from 13 T-ALL samples from the four preclinical trial arms with each lanes representing one animal. Dots on the left indicate blot identities. Arrows on the right indicate bands that represent specific phospho-signals.

We next sub-lethally irradiated a cohort of congenic mice and transplanted them with JW-81, an aggressive primary T-ALL. JW-81 was generated in wild-type mice, harbors Moloney leukemia virus insertions in *Rasgrp1* and other genes, and has been investigated in previous preclinical trials [25]. We selected T-ALL JW81 as a model system for testing the hypothesis that GDC0941 and AS-605 would demonstrate synergistic anti-leukemia activity in *vivo*. T-ALL JW81 is an aggressive leukemia that causes lethality in transplant recipients after ~15 days characterized by high blood leukocyte counts and extensive proliferation of leukemic blasts in the bone marrow that also invade the central nervous system (CNS) [25]. Four days after irradiation and transplantation, recipient mice were randomized into four treatment arms: vehicle (hydroxypropyl methylcellulose) control, GDC0941 alone, AS-605 alone, or combination treatment with GDC0941 and AS-606 (**Fig 6A**). Mice were treated daily via oral gavage until they required euthanasia for progressive leukemia. Consistent with previous data [25], GDC0941 modestly extended survival (green line) compared to vehicle-treated mice (black line). Mice treated with AS-605 alone (purple line) demonstrated a trend towards an intermediate response to treatment. However, the GDC0941/AS-605 combination did not replicate the synergistic effects observed in vitro (**Fig 6B**). The rapid morbidity observed in the GDC0941/AS-605 cohort is unlikely caused by drug toxicity as daily administration of GDC0941 (100 mg/kg/day) combined with AS-605 (50 mg/kg/day) for four weeks in the MTD assessment did not lead to obvious toxicity (**S2 Fig**). Southern blot analysis of refractory T-ALL cells isolated at euthanasia showed no change in the pattern of viral integrations (**Fig 6C**), which is indicative of clonal evolution and can be a marker of intrinsic drug resistance [25, 32, 33]. Finally, AS-605 did not enhance the efficacy of GDC0941 in a second cohort of recipient mice that received a slightly modified regimen of 125 mg/kg GDC0941 every day and 50 mg/kg AS-605 on five days out of the week (**S3 Fig)**.

We had previously observed that PI3K-γ and δ inhibition is more effective at suppressing inflammatory signatures than full pan-PI3K inhibition [34]. We explored the bone marrow samples from the first cohort to investigate our preclinical trial in more detail. Southern blot analysis of refractory T-ALL cells isolated at euthanasia showed no change in the pattern of viral integrations (**Fig 6C**), which is indicative of clonal evolution and can be a marker of intrinsic drug resistance [25, 32, 33]. Based on these data, drug resistant JW-81 cells did not appear to result from selection and outgrowth of a clone with distinct genetic features.

To investigate variability or plasticity in biochemical signaling pathways as a potential explanation for the response of T-ALL JW81 to the GDC0941/AS-605 combination, we subjected the same bone marrow isolated from mice enrolled on the preclinical trial to biochemical analysis. Analysis of Ras signaling molecules including RasGRP1, SOS1, and Ras itself revealed that levels were roughly equal in most samples without evidence of up- or down-regulation in response to drug treatment (**Fig 6D**). Two observations were made as a result of Western blotting for pAkt, pS6, pERK, and pIκB as a measurement of kinase pathway output. First, bone marrow samples from the GDC0941/AS-605 arm did not reveal high pAkt and pS6 levels, so there was no hyperactivation of PI3K signaling. Secondly, we observed a tremendous level of overall variability in kinase pathway-use without obvious patterns between treatment arms or even within a given arm. This level of variation or plasticity was further underscored by analysis of cMyc and P21^CIP^ levels (**Fig 6D**). In sum, GDC0941/AS-605 combination therapy did not lead to the anticipated additive or synergistic effects *in vivo* or drive outgrowth of genetically distinct T-ALL subclones.

## Discussion

There is a need to improve T-ALL therapy, especially for relapsing patients as their prognosis is dismal and treatment options are very limited. Genetic [17] and biochemical [6, 18, 20, 21] studies motivated us to systematically analyze the PI3K/Akt pathway using multiplex, multiparameter flow cytometry and pan- and isoform specific-PI3K inhibitor treatment in T-ALL. We find that the PI3K/Akt pathway is frequently active in T-ALL cells but with very heterogeneous patterns, that pan-PI3K but also γ-specific inhibitors can efficiently suppress biochemical activation of Akt in T-ALL cells, and that combination therapy including PI3K inhibition is a promising direction but requires high-resolution investigations to reveal mechanistic insights in order to bring these treatment strategies into the clinic.

There is an ongoing interest in isoform-specific PI3K inhibitors as a result of emerging evidence that different tumors depend on distinct PI3K isoforms and thus isoform-specific inhibitors may yield therapeutic effects without causing general cytotoxicity [27, 35]. The AS-605 PI3Kγ inhibitor achieved efficient biochemical inhibition of PI3K-Akt signaling in all our T-ALL cell lines but this did not translate into cytotoxic effects *in vitro*. The same result was observed after treatment with the pan-PI3K inhibitor GDC0941. Similarly, only cytostatic effects of pan-PI3K inhibition have been observed in other cancer types [36–38]. The PI3Kγ inhibitor AS-605 had only very modest effects in our preclinical trial studies using mice transplanted with primary JW-81 T-ALL cells. These data are in agreement with previous studies demonstrating that genetic deletion of both PI3Kγ and δ in PTEN-deficient T-ALLs significantly extends survival of mice, whereas deletion of PI3Kγ alone has very little effect [26]. Thus, inhibition of PI3Kγ alone does not appear sufficient for T-ALL therapy, despite measurable inhibition of PI3K-Akt signaling. This does not indicate that PI3Kγ inhibition will not represent a useful treatment strategy in other contexts. In fact, it was recently reported that PI3Kγ inhibition drives macrophages into more inflammatory programs and appears to synergize with cancer immune-therapy [39].

Combining PI3K inhibitors with compounds that block other pathways and/or standard of care chemotherapy drugs is a logical approach for T-ALL treatment [40–42], but the rules that govern potential efficacy of combined pathway inhibition are unclear. Combinatorial inhibition of PI3Kγ and δ efficiently reduced the growth of PTEN-deficient T-ALLs [26]. We observed strong synergy *in vitro* when combining the AS-605 PI3Kγ inhibitor with the pan-PI3K inhibitor GDC0941. Surprisingly, combining these inhibitors *in vivo* provided no survival benefit in our preclinical studies. These findings are reminiscent of our report demonstrating that combined PI3Kγ and δ inhibition is more effective at suppressing inflammatory signatures than full pan-PI3K inhibition [34], and argue that the potential effects of these combination therapies should be carefully examined. For example, it’s possible that combined treatment with AS-605 and GDC0941 perturbs an unknown PI3K-dependent negative feedback loop that normally functions to dampen T-ALL growth, and that this loop remains intact upon treatment with single agent GDC0941 or combined PI3Kγ and δ inhibition. Our analysis of several signaling pathways in bone marrow cells from mice enrolled on the preclinical trial did not reveal clear patterns of activation or repression. However, traditional biochemical analyses only provide mere snapshots of complex signaling pathways in cancer cells at the population rather than the single-cell level. We anticipate that these studies will benefit from future efforts with high-dimensional, high-resolution technologies such as CyTOF to resolve the biochemical complexity at a single-cell level [43].

## Materials and methods

### Cell lines

Generation and culturing conditions for all murine and human cell lines was described previously in [6, 44]. In short, all T-ALL lines were propagated at roughly 1–2 millian cells/ml in RPMI, 10% FBS, penicillin/streptomycin, and β-mercaptoethanol using standard cell culture conditions.

### Antibodies

The following antibodies were used in this study: phospho-Akt (S473) Cell signaling #4058, phospho-Akt (T308) Cell signaling #2965, phospho-p53 Cell signaling #12571, α-tubulin Sigma T6074, p110α Cell signaling #4249, p110β Milipore 05–1558, p110δ Santa Cruz Biotechnologies sc-7176, p110γ Cell signaling #5450, phospho S6 (S235/236) Cell signaling #2211 cleaved caspase 3 Cell signaling #9661, cleaved PARP AlexaFluor 647 BD Pharmingen 558710, donkey anti-rabbit PE (711-116-152) and donkey anti-rabbit APC (711-136-152) from Jackson ImmunoResearch.

### Compounds

AS-605240, AS-252424 and GDC0941 were purchased from Selleckchem; PIK90, PI-103, BH9, TGX221, SW30, SW14, PIK92 and INK128 were gifts from Kevan Shokat laboratory, UCSF. All inhibitors were resuspended in DMSO at 10 mM and used at final concentration of 10 μM, unless otherwise indicated.

### Cytokines stimulations and Western blotting.

Cells were washed and rested in PBS at 37°C for 30 min prior to stimulation. Where indicated cells were treated with DMSO or inhibitors during resting. After resting, cells were stimulated with cytokines: IL-2, IL-7, IL-9 (each at 10 ng/ml unless otherwise noted; Peprotech) and subjected to NP40 lysis preparation and Western blotting, as described previously [44]. For Western blot analysis on samples from mice in preclinical trial, bone marrow was harvested from moribund animals on the trial and cryopreserved in 90% FBS + 10% DMSO. Samples were subsequently thawed from cryopreservation and immediately lysed in ice-cold lysis buffer.

### *In vitro* stimulations, drug treatment and fluorescent barcoding

Cells were washed three times in PBS and 0.4x10^6^ cells in 75 μl were plated into a 96 well plate (round bottom). Cells were rested in the presence of inhibitors (10 μM unless stated otherwise) or vehicle control (DMSO added 1/100) for 30 minutes at 37°C. Next, cells were stimulated with a cocktail of cytokines (final concentrations: IL-2, 20 ng/ml; IL-7, 10 ng/ml; IL-9, 5 ng/ml) for 2, 5 or 10 minutes, then fixed, permeabilized and barcoded as described previously [44]. For barcoding, the following concentrations of dyes were used to achieve best separation of populations: for Alexa Fluor 488–15, 5, 1.3, 0.3, 0.075 and 0.018 μg/ml; for Pacific Blue– 40, 6.5, 0.6 and 0.075 μg/ml. Samples were acquired on a BD LSR II and analyzed with Cytobank.

### Apoptosis assay

Cells were plated into a 96-well plate in full culturing media and drugs were added to the final concentration as indicated. Staurosporine (1μM) was used as a positive control for induction of apoptosis. Cells were incubated with drugs for 8 hours. Next, cells were washed with PBS and fixed with 2% PFA for 20 minutes at room temperature (RT). After washing with FACS buffer, cells were resuspended in 90% ice-cold methanol and permeabilized overnight at -20°C. Next, cells were washed and stained for cleaved caspase-3 and cleaved PARP conjugated to Alexa Fluor 647 followed by secondary antibody staining with donkey anti-rabbit PE. Samples were run on a BD LSR II or Fortessa and analyzed with FlowJO.

### Proliferation assay

Cell proliferation was determined by measuring dilution of CFSE (Invitrogen C1157) by flow cytometry. Cells were stained with CFSE according to the manufacturer’s protocol, and 2 μM CFSE was used as the final concentration. Cells were plated into 6 well plates in duplicate for each condition (multiple concentrations of inhibitors were tested). Samples were collected at 16, 44 and 68 hours, stained with DAPI to distinguish live (DAPI negative) and dead (DAPI positive) cells and acquired on a BD LSR II. Proliferation index was calculated by dividing geometric mean of DMSO-treated sample by geometric mean of inhibitor-treated sample.

### Gene expression analysis in pediatric T-ALL patients

Patient sample collection and p110 isoform expression analysis was performed as described previously [6]. In all cases multiple probe sets were identified for the gene of interest. Data were normalized using RMA.

### Growth assay

Cells in full culturing media were plated into 24-well plates in duplicate together with the indicated drugs and cultured for 3 or 4 days. Water or DMSO was used as a vehicle control. Subsequently, cells were counted using a Muse Cell Analyzer (Merck Milipore). Growth rate was calculated by dividing cell numbers at the end of the experiment by the number of cells at day 0. Growth expressed as a percentage was obtained by normalizing to a control that was set as 100%.

### Determining maximum tolerated dose and preclinical trial

C57Bl6/129SV (FBV) mice were sublethally irradiated and treatment with vehicle (Hydroxylpropyl methylcellulose), GDC0941, AS-605 or the combination (3 mice per group) was started 4 days later. Vehicle and all drugs were administered daily by oral gavage. After 4 weeks of treatment mice were euthanized, a general examination of organs was performed, and a complete blood count (CBC) was obtained for one mouse from each group. No obvious signs of toxicity were observed. For the preclinical trial, mice were sublethally irradiated and injected with 2x10^6^ freshly harvested JW-81 T-ALL cells via the tail vein. Treatment was started 4 days post-transplant and continued until progressive disease required euthanasia. The trial was performed with AS-605 at 50 mg/kg, GDC0941 at 100 mg/kg or a combination of the two drugs at these concentrations. Three mice were included in the vehicle group and 5 mice were included for each treatment arm. Mice were monitored for signs of leukemic progression, and euthanized when they became moribund.

### Southern blotting

Southern blot analysis was performed to analyze the patterns of leukemia virus insertions in JW-81 T-ALL. Genomic DNA was isolated from bone marrow that was harvested from moribund animals on the trial and Southern blots were run and hybridized as described in [30] with the exception that a Random Primer DNA Labeling Kit Ver.2 (Cat# 6045) from Takara Biotechnology, Inc. was used to label the MOL4070 LTR probe.

## Supporting information

S1 Fig Expression of RasGRP1, PI3K isoforms and clinical outcome. A. Graphs show correlation analysis between expression of RasGRP1 and different PI3K isoforms (mRNA levels) in over 100 T-ALL patients from 9404 and AALL0434 studies. To determine correlation between two continuous variables Pearson’s correlation coefficient (r) was calculated. B. Comparison of RasGRP1 expression (mRNA levels) in patients who underwent complete remission (CR) or Induction Failure (IF). p = 0.0001 (t-test) C. Comparison of different PI3K isoforms expression (mRNA levels) in patients who underwent complete remission or induction failure. Significance determined by t test.
(TIF)

S2 Fig Maximum tolerated dose (MTD) trial for GDC0941, AS-605 and combination of the two drugs. A. Schematic illustration of a trial testing MTD. Mice were sublethally irradiated and after 4 days were randomized into 6 treatment arms (3 mice per group): vehicle control, GDC0941 (100 mg/kg), AS-605 50 mg/kg, GDC0941 (100 mg/kg) + AS-605 50 mg/kg, GDC0941 (100 mg/kg)+ AS-605 30 mg/kg and GDC0941 (100 mg/kg)+ AS-605 10 mg/kg. Drugs were given daily for 4 weeks via oral gavage. Subsequently, mice were euthanized and their organs examined for general toxicity. Complete blood count (CBC) was run for one of the mouse from each treatment group. B. Table showing CBC results for individual mice on the MTD trial. WBC = white blood count [K/μl]; Hb = hemoglobin [g/μl]; PLT = platelets [K/μl].
(TIF)

S3 Fig Combining GDC0941 and AS-605 inhibitors does not show *in vivo* efficacy. A. Diagram showing preclinical trial set-up. Four days after transplant of primary JW-81 T-ALL cells, mice were randomized into four treatment arms: vehicle control, GDC0941, AS-605, or GDC0941 + AS-605. Three mice were enrolled on the vehicle control arm and five mice were enrolled on each treatment arm. In this trial GDC0941 and AS-605 were given at different timepoints and in different regimen. GDC0941 was given at 125mg/kg every day of the week (Monday through Sunday). AS-605 was given at 50 mg/kg on five days of the week, 3 hours after GDC0941 administration (Monday through Friday). **B.** Kaplan-Meier graph showing percentage of mice surviving treatment with PI3K inhibitors as a function of time.
(TIF)

## References

[1] Pui C, Evans W. Treatment of acute lymphoblastic leukemia. N Engl J Med. 2006;354(2):166–78. doi: 354/2/166 [pii] doi: 10.1056/NEJMra052603 16407512 10.1056/NEJMra052603

[2] Inaba H, Pui CH. Glucocorticoid use in acute lymphoblastic leukaemia. Lancet Oncol. 11(11):1096–106. Epub 2010/10/16. doi: S1470-2045(10)70114-5 [pii] doi: 10.1016/S1470-2045(10)70114-5 . 20947430 10.1016/S1470-2045(10)70114-5 PMC3309707

[3] Aifantis I, Raetz E, Buonamici S. Molecular pathogenesis of T-cell leukaemia and lymphoma. Nature Reviews Immunology. 2008;8(5):380–90. doi: 10.1038/nri2304 18421304 10.1038/nri2304

[4] DeAngelo DJ. A phase I clinical trial of the notch inhibitor MK-0752 in patients with T-cell acute lymphoblastic leukemia (T-ALL) and other leukemias. Journal of Clinical Oncology 2006;2006 ASCO Annual Meeting Proceedings, 24, 6585.

[5] von Lintig FC, Huvar I, Law P, Diccianni MB, Yu AL, Boss GR. Ras activation in normal white blood cells and childhood acute lymphoblastic leukemia. Clinical Cancer Research. 2000;6(5):1804–10. 10815901

[6] Hartzell C, Ksionda O, Lemmens E, Coakley K, Yang M, Dail M, et al Dysregulated RasGRP1 Responds to Cytokine Receptor Input in T Cell Leukemogenesis. Sci Signal. 2013;6(268):ra21 doi: 10.1126/scisignal.2003848 23532335 10.1126/scisignal.2003848 PMC3737252

[7] Ksionda O, Limnander A, Roose JP. RasGRP Ras guanine nucleotide exchange factors in cancer. Front Biol (Beijing). 2013;8(5):508–32. doi: 10.1007/s11515-013-1276-9 ; PubMed Central PMCID: PMC3987922. 24744772 10.1007/s11515-013-1276-9 PMC3987922

[8] Oki T, Kitaura J, Watanabe-Okochi N, Nishimura K, Maehara A, Uchida T, et al Aberrant expression of RasGRP1 cooperates with gain-of-function NOTCH1 mutations in T-cell leukemogenesis. Leukemia. 2011 doi: 10.1038/leu.2011.328 22116551 10.1038/leu.2011.328

[9] Vivanco I, Sawyers CL. The phosphatidylinositol 3-Kinase AKT pathway in human cancer. Nat Rev Cancer. 2002;2(7):489–501. doi: 10.1038/nrc839 . 12094235 10.1038/nrc839

[10] Downward J. Cancer biology: signatures guide drug choice. Nature. 2006;439(7074):274–5. doi: 10.1038/439274a . 16421553 10.1038/439274a

[11] Schubbert S, Shannon K, Bollag G. Hyperactive Ras in developmental disorders and cancer. Nat Rev Cancer. 2007;7(4):295–308. doi: 10.1038/nrc2109 17384584 10.1038/nrc2109

[12] Vanhaesebroeck B, Stephens L, Hawkins P. PI3K signalling: the path to discovery and understanding. Nature Publishing Group. 2012;13(3):195–203. doi: 10.1038/nrm3290 22358332 10.1038/nrm3290

[13] Gupta S, Ramjaun AR, Haiko P, Wang Y, Warne PH, Nicke B, et al Binding of ras to phosphoinositide 3-kinase p110alpha is required for ras-driven tumorigenesis in mice. Cell. 2007;129(5):957–68. doi: 10.1016/j.cell.2007.03.051 . 17540175 10.1016/j.cell.2007.03.051

[14] Fritsch R, de Krijger I, Fritsch K, George R, Reason B, Kumar MS, et al RAS and RHO families of GTPases directly regulate distinct phosphoinositide 3-kinase isoforms. Cell. 2013;153(5):1050–63. Epub 2013/05/28. doi: S0092-8674(13)00506-0 [pii] doi: 10.1016/j.cell.2013.04.031 . 23706742 10.1016/j.cell.2013.04.031 PMC3690480

[15] Burke JE, Williams RL. Synergy in activating class I PI3Ks. Trends Biochem Sci. 2015;40(2):88–100. Epub 2015/01/13. doi: S0968-0004(14)00220-5 [pii] doi: 10.1016/j.tibs.2014.12.003 . 25573003 10.1016/j.tibs.2014.12.003

[16] Kang S, Bader AG, Zhao L, Vogt PK. Mutated PI 3-kinases: cancer targets on a silver platter. Cell Cycle. 2005;4(4):578–81. Epub 2005/05/07. doi: 1593 [pii] doi: 10.4161/cc.4.4.1586 . 15876869 10.4161/cc.4.4.1586

[17] Gutierrez A, Sanda T, Grebliunaite R, Carracedo A, Salmena L, Ahn Y, et al High frequency of PTEN, PI3K, and AKT abnormalities in T-cell acute lymphoblastic leukemia. Blood. 2009;114(3):647–50. doi: 10.1182/blood-2009-02-206722 19458356 10.1182/blood-2009-02-206722 PMC2713461

[18] Silva A, Yunes JA, Cardoso BA, Martins LR, Jotta PY, Abecasis M, et al PTEN posttranslational inactivation and hyperactivation of the PI3K/Akt pathway sustain primary T cell leukemia viability. Journal of Clinical Investigation. 2008;118(11):3762–74. doi: 10.1172/JCI34616 18830414 10.1172/JCI34616 PMC2556239

[19] Maser RS, Choudhury B, Campbell PJ, Feng B, Wong K-K, Protopopov A, et al Chromosomally unstable mouse tumours have genomic alterations similar to diverse human cancers. Nature. 2007;447(7147):966–71. doi: 10.1038/nature05886 17515920 10.1038/nature05886 PMC2714968

[20] Palomero T, Sulis M, Cortina M, Real P, Barnes K, Ciofani M, et al Mutational loss of PTEN induces resistance to NOTCH1 inhibition in T-cell leukemia. Nat Med. 2007;13(10):1203–10. doi: nm1636 [pii] doi: 10.1038/nm1636 17873882 10.1038/nm1636 PMC2600418

[21] Lonetti A, Antunes IL, Chiarini F, Orsini E, Buontempo F, Ricci F, et al Activity of the pan-class I phosphoinositide 3-kinase inhibitor NVP-BKM120 in T-cell acute lymphoblastic leukemia. Leukemia. 2014;28(6):1196–206. Epub 2013/12/07. doi: leu2013369 [pii] doi: 10.1038/leu.2013.369 . 24310736 10.1038/leu.2013.369

[22] Chiarini F, Fala F, Tazzari PL, Ricci F, Astolfi A, Pession A, et al Dual Inhibition of Class IA Phosphatidylinositol 3-Kinase and Mammalian Target of Rapamycin as a New Therapeutic Option for T-Cell Acute Lymphoblastic Leukemia. Cancer Research. 2009;69(8):3520–8. doi: 10.1158/0008-5472.CAN-08-4884 19351820 10.1158/0008-5472.CAN-08-4884 PMC3836286

[23] Bressanin D, Evangelisti C, Ricci F, Tabellini G, Chiarini F, Tazzari PL, et al Harnessing the PI3K/Akt/mTOR pathway in T-cell acute lymphoblastic leukemia: eliminating activity by targeting at different levels. Oncotarget. 2012;3(8):811–23. Epub 2012/08/14. doi: 579 [pii] doi: 10.18632/oncotarget.579 . 22885370 10.18632/oncotarget.579 PMC3478458

[24] Lonetti A, Cappellini A, Sparta AM, Chiarini F, Buontempo F, Evangelisti C, et al PI3K pan-inhibition impairs more efficiently proliferation and survival of T-cell acute lymphoblastic leukemia cell lines when compared to isoform-selective PI3K inhibitors. Oncotarget. 2015;6(12):10399–414. Epub 2015/04/15. doi: 3295 [pii] doi: 10.18632/oncotarget.3295 . 25871383 10.18632/oncotarget.3295 PMC4496363

[25] Dail M, Wong J, Lawrence J, O'Connor D, Nakitandwe J, Chen SC, et al Loss of oncogenic Notch1 with resistance to a PI3K inhibitor in T-cell leukaemia. Nature. 2014;513(7519):512–6. doi: 10.1038/nature13495 . 25043004 10.1038/nature13495 PMC4213126

[26] Subramaniam PS, Whye DW, Efimenko E, Chen J, Tosello V, De Keersmaecker K, et al Targeting Nonclassical Oncogenes for Therapy in T-ALL. Cancer Cell. 2012;21(4):459–72. doi: 10.1016/j.ccr.2012.02.029 22516257 10.1016/j.ccr.2012.02.029

[27] Vanhaesebroeck B, Guillermet-Guibert J, Graupera M, Bilanges B. The emerging mechanisms of isoform-specific PI3K signalling. Nat Rev Mol Cell Biol. 2010;11(5):329–41. doi: 10.1038/nrm2882 20379207 10.1038/nrm2882

[28] Sarbassov DD, Guertin DA, Ali SM, Sabatini DM. Phosphorylation and regulation of Akt/PKB by the rictor-mTOR complex. Science. 2005;307(5712):1098–101. Epub 2005/02/19. doi: 307/5712/1098 [pii] doi: 10.1126/science.1106148 . 15718470 10.1126/science.1106148

[29] Manning BD, Cantley LC. AKT/PKB signaling: navigating downstream. Cell. 2007;129(7):1261–74. doi: 10.1016/j.cell.2007.06.009 17604717 10.1016/j.cell.2007.06.009 PMC2756685

[30] Dail M, Li Q, McDaniel A, Wong J, Akagi K, Huang B, et al Mutant Ikzf1, KrasG12D, and Notch1 cooperate in T lineage leukemogenesis and modulate responses to targeted agents. Proceedings of the National Academy of Sciences. 2010;107(11):5106–11. doi: 10.1073/pnas.1001064107 20194733 10.1073/pnas.1001064107 PMC2841878

[31] Camps M, Ruckle T, Ji H, Ardissone V, Rintelen F, Shaw J, et al Blockade of PI3Kgamma suppresses joint inflammation and damage in mouse models of rheumatoid arthritis. Nat Med. 2005;11(9):936–43. Epub 2005/08/30. doi: 10.1038/nm1284 . 16127437 10.1038/nm1284

[32] Lauchle JO, Kim D, Le DT, Akagi K, Crone M, Krisman K, et al Response and resistance to MEK inhibition in leukaemias initiated by hyperactive Ras. Nature. 2009;461(7262):411–4. doi: 10.1038/nature08279 19727076 10.1038/nature08279 PMC4119783

[33] Burgess MR, Hwang E, Firestone AJ, Huang T, Xu J, Zuber J, et al Preclinical efficacy of MEK inhibition in Nras-mutant AML. Blood. 2014;124(26):3947–55. Epub 2014/11/02. doi: blood-2014-05-574582 [pii] doi: 10.1182/blood-2014-05-574582 . 25361812 10.1182/blood-2014-05-574582 PMC4271180

[34] Williams O, Houseman BT, Kunkel EJ, Aizenstein B, Hoffman R, Knight ZA, et al Discovery of dual inhibitors of the immune cell PI3Ks p110delta and p110gamma: a prototype for new anti-inflammatory drugs. Chem Biol. 2010;17(2):123–34. Epub 2010/03/02. doi: S1074-5521(10)00038-4 [pii] doi: 10.1016/j.chembiol.2010.01.010 . 20189103 10.1016/j.chembiol.2010.01.010 PMC2858875

[35] Thorpe LM, Yuzugullu H, Zhao JJ. PI3K in cancer: divergent roles of isoforms, modes of activation and therapeutic targeting. Nat Rev Cancer. 2015;15(1):7–24. Epub 2014/12/24. doi: nrc3860 [pii] doi: 10.1038/nrc3860 . 25533673 10.1038/nrc3860 PMC4384662

[36] Garcia-Martinez JM, Wullschleger S, Preston G, Guichard S, Fleming S, Alessi DR, et al Effect of PI3K- and mTOR-specific inhibitors on spontaneous B-cell follicular lymphomas in PTEN/LKB1-deficient mice. Br J Cancer. 104(7):1116–25. Epub 2011/03/17. doi: bjc201183 [pii] doi: 10.1038/bjc.2011.83 . 21407213 10.1038/bjc.2011.83 PMC3068512

[37] Martini M, Ciraolo E, Gulluni F, Hirsch E. Targeting PI3K in Cancer: Any Good News? Front Oncol. 3:108 Epub 2013/05/10. doi: 10.3389/fonc.2013.00108 . 23658859 10.3389/fonc.2013.00108 PMC3647219

[38] Gautam P, Karhinen L, Szwajda A, Jha SK, Yadav B, Aittokallio T, et al Identification of selective cytotoxic and synthetic lethal drug responses in triple negative breast cancer cells. Molecular Cancer. 2016;15(1):34 doi: 10.1186/s12943-016-0517-3 27165605 10.1186/s12943-016-0517-3 PMC4862054

[39] Kaneda MM, Messer KS, Ralainirina N, Li H, Leem CJ, Gorjestani S, et al PI3Kgamma is a molecular switch that controls immune suppression. Nature. 2016;539(7629):437–42. Epub 2016/11/01. doi: nature19834 [pii] doi: 10.1038/nature19834 . 27642729 10.1038/nature19834 PMC5479689

[40] Wallin JJ, Guan J, Prior WW, Lee LB, Berry L, Belmont LD, et al GDC-0941, a novel class I selective PI3K inhibitor, enhances the efficacy of docetaxel in human breast cancer models by increasing cell death in vitro and in vivo. Clin Cancer Res. 2012;18(14):3901–11. Epub 2012/05/16. doi: 1078-0432.CCR-11-2088 [pii] doi: 10.1158/1078-0432.CCR-11-2088 . 22586300 10.1158/1078-0432.CCR-11-2088

[41] Floris G, Wozniak A, Sciot R, Li H, Friedman L, Van Looy T, et al A potent combination of the novel PI3K Inhibitor, GDC-0941, with imatinib in gastrointestinal stromal tumor xenografts: long-lasting responses after treatment withdrawal. Clin Cancer Res. 2013;19(3):620–30. Epub 2012/12/13. doi: 1078-0432.CCR-12-2853 [pii] doi: 10.1158/1078-0432.CCR-12-2853 . 23231951 10.1158/1078-0432.CCR-12-2853 PMC4405777

[42] Munugalavadla V, Mariathasan S, Slaga D, Du C, Berry L, Del Rosario G, et al The PI3K inhibitor GDC-0941 combines with existing clinical regimens for superior activity in multiple myeloma. Oncogene. 2014;33(3):316–25. Epub 2013/01/16. doi: onc2012594 [pii] doi: 10.1038/onc.2012.594 . 23318440 10.1038/onc.2012.594

[43] Spitzer MH, Nolan GP. Mass Cytometry: Single Cells, Many Features. Cell. 2016;165(4):780–91. Epub 2016/05/08. doi: S0092-8674(16)30410-X [pii] doi: 10.1016/j.cell.2016.04.019 . 27153492 10.1016/j.cell.2016.04.019 PMC4860251

[44] Ksionda O, Melton AA, Bache J, Tenhagen M, Bakker J, Harvey R, et al RasGRP1 overexpression in T-ALL increases basal nucleotide exchange on Ras rendering the Ras/PI3K/Akt pathway responsive to protumorigenic cytokines. Oncogene. 2015;35(28):3658–68. Epub 2015/11/10. doi: onc2015431 [pii] doi: 10.1038/onc.2015.431 . 26549032 10.1038/onc.2015.431 PMC4868787

