# Supplementary figures and images for: Comprehensive analysis of T cell leukemia signals reveals heterogeneity in the PI3 kinase-Akt pathway and limitations of PI3 kinase inhibitors as monotherapy
Source: PLoS One. 2018 May 25;13(5):e0193849. doi: 10.1371/journal.pone.0193849 (PMC5969748; doi:10.1371/journal.pone.0193849)

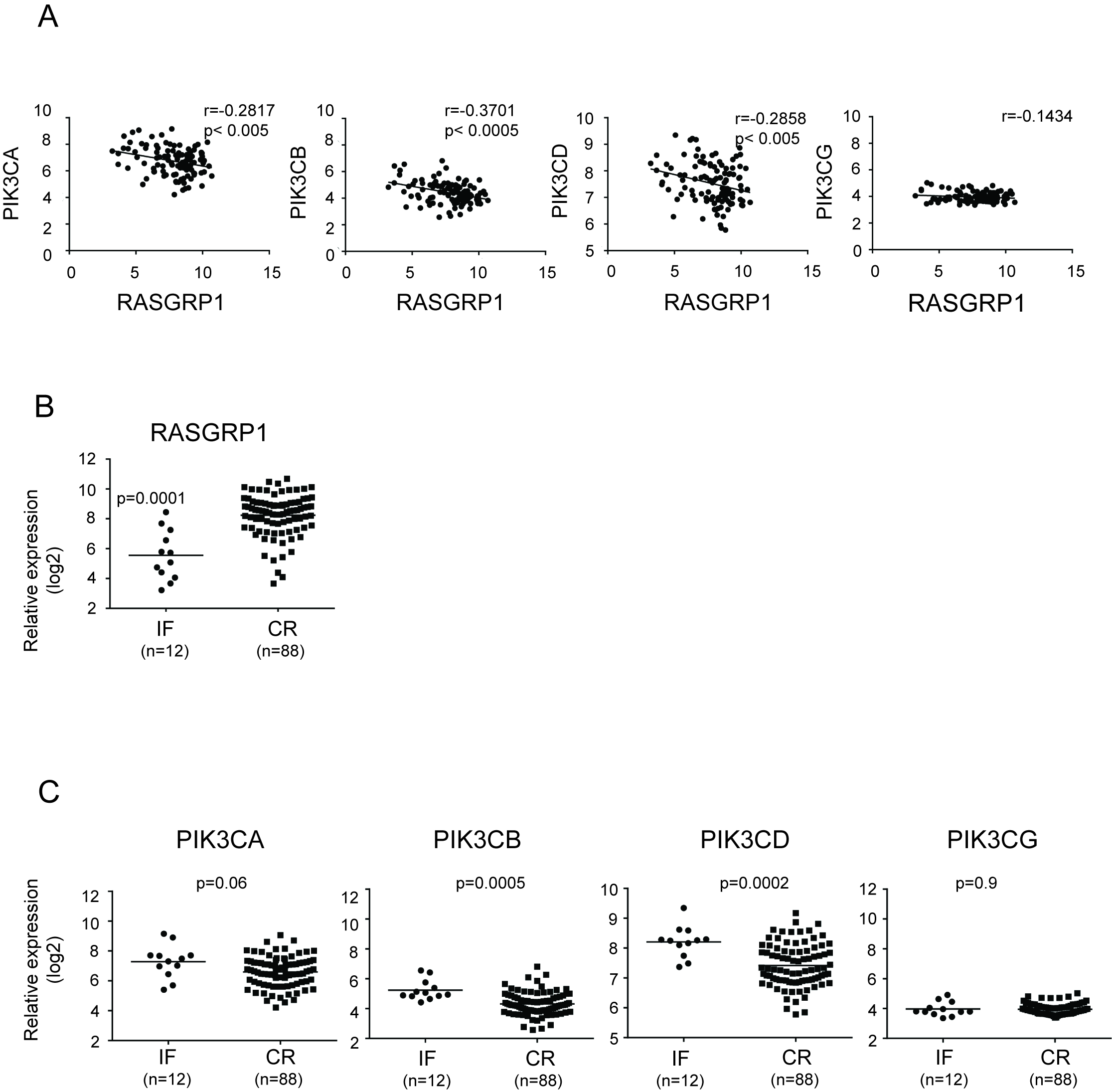

Supplement: S1 Fig — A. Graphs show correlation analysis between expression of RasGRP1 and different PI3K isoforms (mRNA levels) in over 100 T-ALL patients from 9404 and AALL0434 studies. To determine correlation between two continuous variables Pearson’s correlation coefficient (r) was calculated. B. Comparison of RasGRP1 expression (mRNA levels) in patients who underwent complete remission (CR) or Induction Failure (IF). p = 0.0001 (t-test) C. Comparison of different PI3K isoforms expression (mRNA levels) in patients who underwent complete remission or induction failure. Significance determined by t test. (TIF) [file pone.0193849.s001.tif]

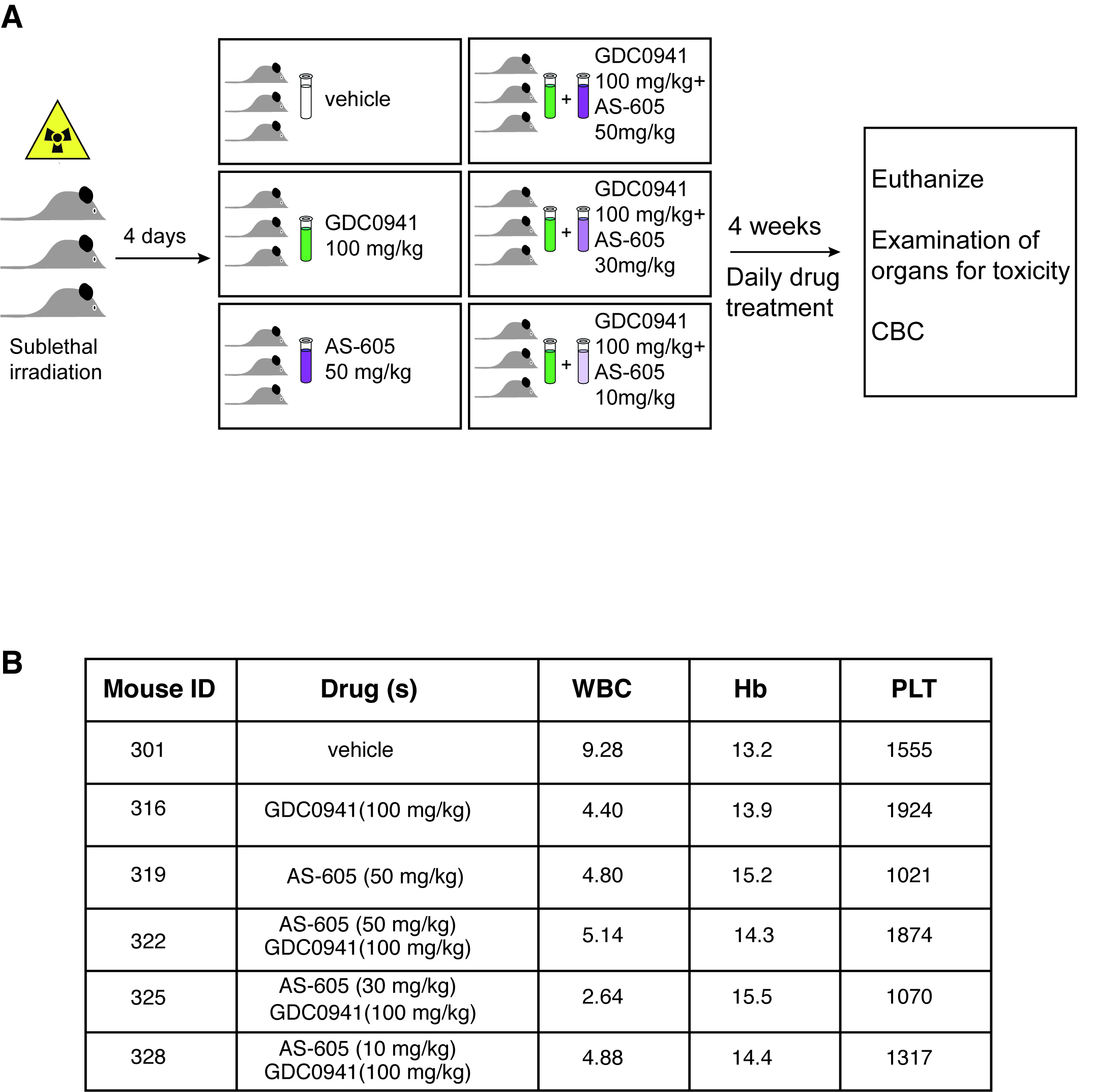

Supplement: S2 Fig — A. Schematic illustration of a trial testing MTD. Mice were sublethally irradiated and after 4 days were randomized into 6 treatment arms (3 mice per group): vehicle control, GDC0941 (100 mg/kg), AS-605 50 mg/kg, GDC0941 (100 mg/kg) + AS-605 50 mg/kg, GDC0941 (100 mg/kg)+ AS-605 30 mg/kg and GDC0941 (100 mg/kg)+ AS-605 10 mg/kg. Drugs were given daily for 4 weeks via oral gavage. Subsequently, mice were euthanized and their organs examined for general toxicity. Complete blood count (CBC) was run for one of the mouse from each treatment group. B. Table showing CBC results for individual mice on the MTD trial. WBC = white blood count [K/μl]; Hb = hemoglobin [g/μl]; PLT = platelets [K/μl]. (TIF) [file pone.0193849.s002.tif]

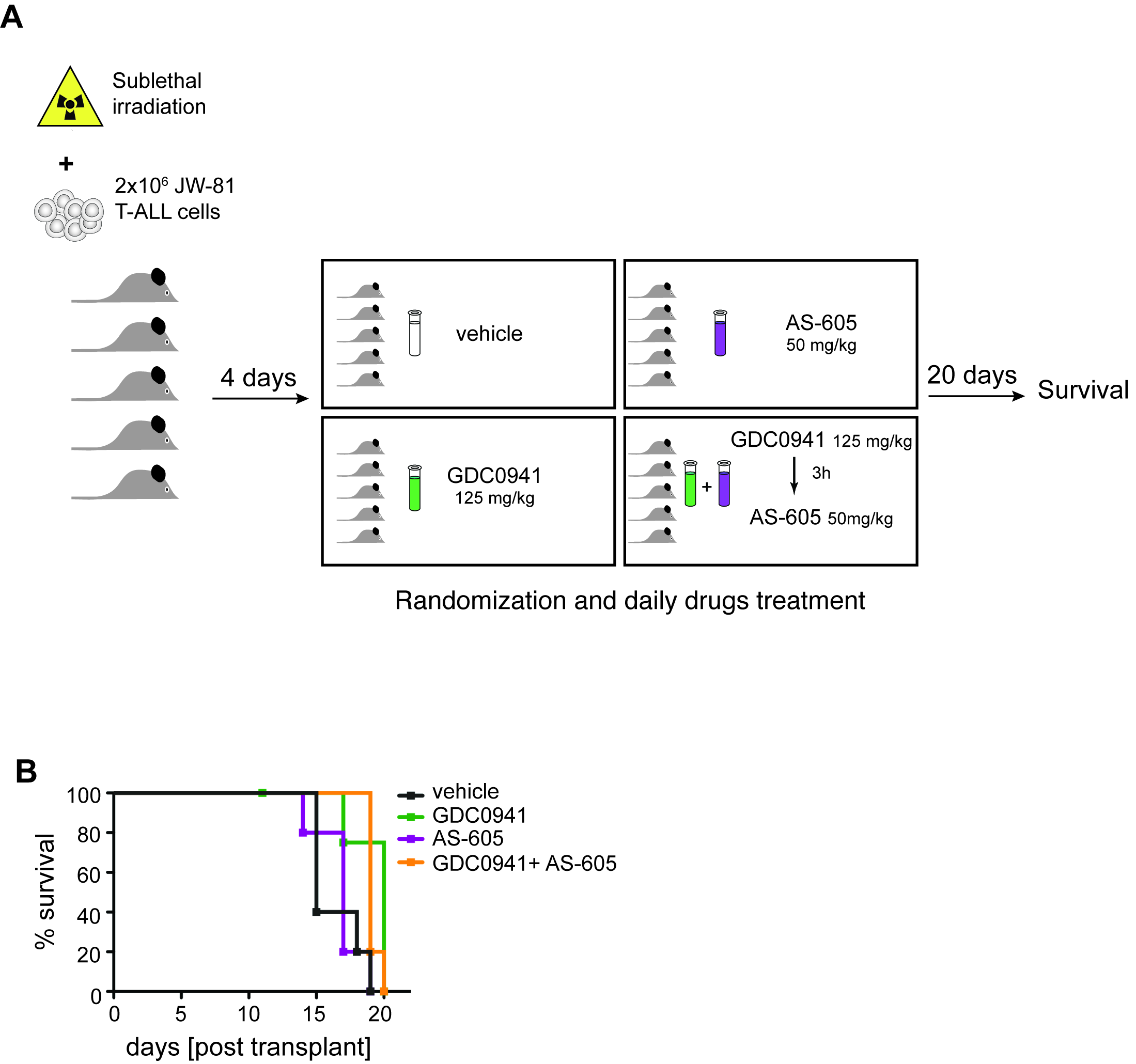

Supplement: S3 Fig — Diagram showing preclinical trial set-up. Four days after transplant of primary JW-81 T-ALL cells, mice were randomized into four treatment arms: vehicle control, GDC0941, AS-605, or GDC0941 + AS-605. Three mice were enrolled on the vehicle control arm and five mice were enrolled on each treatment arm. In this trial GDC0941 and AS-605 were given at different timepoints and in different regimen. GDC0941 was given at 125mg/kg every day of the week (Monday through Sunday). AS-605 was given at 50 mg/kg on five days of the week, 3 hours after GDC0941 administration (Monday through Friday). B. Kaplan-Meier graph showing percentage of mice surviving treatment with PI3K inhibitors as a function of time. (TIF) [file pone.0193849.s003.tif]
